# Comparative performance of hrHPV testing and PAX1/ZNF671 methylation in triaging women with abnormal cytology: a study of paired urine, vaginal and cervical scrape samples

**DOI:** 10.3389/fonc.2026.1765689

**Published:** 2026-03-11

**Authors:** Yuanyuan Wang, Lufang Zhang, Hongke Zhao, Derong Guo, Huimin Guo, Mengwei Miao, Haixia Qin, Ying Liu

**Affiliations:** 1 Department of Gynecology, The First Affiliated Hospital of Henan Medical University, Weihui, Henan, China; 2 Cancer Research Institute/Department of Oncology, Cancer Hospital, The First Affiliated Hospital of Henan Medical University, Weihui, China; 3 Department of Integrative Medicine, The First Affiliated Hospital of Henan Medical University, Weihui, Henan, China; 4 Gynecology Outpatient Department, The First Affiliated Hospital of Henan Medical University, Weihui, Henan, China

**Keywords:** ASC-US, cervical cancer, high-risk human papilloma virus, methylation, Pax1, ZNF671

## Abstract

**Background:**

The purpose was to evaluate the diagnostic effectiveness of High-risk human papillomavirus(hrHPV) testing, DNA methylation, PAX1/ZNF671 methylation in triaging patients with abnormal cytology and/or abnormal cervical biopsy pathology in cervical cancer screening; And the detection performance of different screening strategies was compared among clinician-taken cervical scrapes and paired self-collected urine and vaginal samples.

**Methods:**

A total of 136 urine-based,137self-collected vaginal and 140 cervical scrapes samples were analyzed. Samples were tested for hrHPV DNA and six methylation markers. Various screening strategies from different samples were compared under the definitive histopathology for their diagnostic accuracy against two standards: cervical intraepithelial neoplasia grade 2 or more severe lesions (CIN2+) and cervical intraepithelial neoplasia grade 3 or more severe lesions (CIN3+).

**Results:**

For PAX1 and ZNF671, the areas under the ROC curve were 0.929 and 0.862 and the methylation-positive rate was 89.5% (77/86) and 80.2% (69/86) in CIN3+ lesions. The cutoff values were 7.95and 10.92, respectively, with the highest Youden index values being 0.763 and 0.684, respectively. The sensitivity of hrHPV testing for CIN2+ was 89.80% in cervical scrape to 92.63% in self-collected vaginal samples. And the optimal marker panel (PAX1/ZNF671) resulted in an 85.8% sensitivity and 5.8 PLR for CIN2+ detection in cervical scrapes, and reached a highest sensitivity for CIN3+ in cervical scrapes (91.9%), markedly exceeding that of vaginal (71.4%) and urine (48.1%) samples (P<0.001). The Negative Predictive Value (NPV) for cervical scrapes (85.7%) was higher than self-collected alternatives (P<0.001). As for hrHPV and DNA Methylation, a perfect sensitivity (96.51%), NPV (90.00%) and Negative Likelihood Ratio(NLR) (0.07) for CIN3+ were reached. For hrHPV negative population, trough PAX1/ZNF671 detection, Cervical scrapes showed a highest sensitivity (100.00%) and specificity (91.30%), a 77.78% PPV and 100% NPV were achieved for discriminating CIN3 +.

**Conclusion:**

The prevalence of methylation for PAX1/ZNF671 genes exhibited a strong positive correlation with the severity of cervical lesions, and demonstrates a better diagnostic value for CIN2+ lesions. DNA methylation testing, especially PAX1/ZNF671 offers a promising strategy to detect CIN2/3 lesions or more serious disease. Cervical samples were the perfect candidates for DNA methylation. Furthermore, PAX1/ZNF671 methylation assays had a strong capacity in screening and excludingCIN2+ lesions among HPV-negative individuals.

## 1 Introduction

Currently, cervical cancer continues to be a major global public health issue, ranked fourth in incidence among women, with a rate of 14.1 per 100,000 in 2022. It is the third leading cause of cancer deaths in females, with a mortality rate of 7.1 per 100,000. In that year, there were 661,021 new cases and 348,189 deaths worldwide due to cervical cancer (1). Among females aged 15 to 44, cervical cancer holds a place in the top three most prevalent cancers across 149 countries, and is also one of the three leading causes of cancer-related mortality in 154 countries. Projections indicate that if the incidence and mortality rates of 2022 persist, the global burden of cervical cancer will rise to 760,082 new diagnoses—representing a 14.8% surge—and 411,035 fatalities, which equates to a 17.8% increase, by the year 2030 (2). Given the heavy disease burden of cervical cancer and its preventable and treatable nature, the World Health Organization (WHO) launched the “Global Strategy to Accelerate the Elimination of Cervical Cancer” in 2020. This strategy announced that 194 countries worldwide would work together to achieve the following goals by 2030:1)90% of girls complete HPV vaccination by the age of 15; 2) 70% of women undergo screening with high-performance testing methods before the ages of 35 and 45; 3) 90% of women diagnosed with cervical disease receive treatment (3). Routine cervical cancer screenings and prompt intervention for pre-cancerous lesions are established as the most effective approaches for the prevention of cervical cancer. Pre-cancerous lesions are characterized by abnormal cellular changes that have not yet progressed to cancer. This category includes historically recognized entities such as High-grade Squamous Intraepithelial Lesion (HSIL) and Adenocarcinoma in Situ (AIS). High-grade precursors, specifically Cervical Intraepithelial Neoplasia Grade 2 (CIN2) and Cervical Intraepithelial Neoplasia Grade 3 (CIN3), can emerge within 3 to 5 years following a high-risk human papillomavirus (hrHPV) infection. The progression to invasive cervical cancer typically requires a longer duration, ranging from 20 to 30 years. This extended timeline provides significant opportunities for intervention through hrHPV screening and Pap smear testing, ultimately aimed at reducing the incidence and mortality rates associated with cervical cancer in Western populations (4). Cervical cancer (CC) screening is one of the most effective approaches to cancer prevention. When paired with widespread HPV vaccination coverage, achieving high participation rates in cervical screening becomes a crucial component of the global initiative aimed at ultimately eradicating CC (5). Persistent infection with HPV is widely recognized as the primary risk factor for cervical cancer. While over 200 distinct HPV genotypes have been identified, the ones of greatest public health significance are high-risk HPV genotypes—specifically HPV 16, 18, 31, 33, 35, 39, 45, 51, 52, 56, 58, and 59 (6). Consequently, the screening strategy for cervical cancer has shifted from the previous cervical cytology-based approach to HPV testing as the first-line screening method, alongside the administration of cervical cancer vaccines for the prevention of this disease (7).

According to literature, women adopted HPV-based cervical screening had a lower invasive cervical cancer risk compared with women adopted cytology, women who were HPV-negative and normal cytology at baseline had invasive cervical cancer risks of 1.3 (95% CI 0.6-2.4) and 9.1 (6.7-11.8) per 100–000 person-years, respectively (8). Although hrHPV DNA screening has also been extensively applied in cervical cancer screening. It exhibits high sensitivity in detecting HPV-associated lesions. However, its low specificity is a major limitation. Since about 90% of HPV infections are transient, a large number of women with positive HPV test results do not actually have precancerous or cancerous lesions, leading to unnecessary anxiety and over-referral for colposcopy (9).

Given the limitations of existing screening methods, there is an urgent need for more accurate and efficient screening strategies. DNA methylation, an important epigenetic modification, has been shown to play a crucial role in the development of cervical cancer. Aberrant methylation of certain genes occurs early in the carcinogenesis process and can serve as potential biomarkers for early detection (10). For instance, methylation of genes such as PAX1 and JAM3 has been associated with the progression of cervical intraepithelial neoplasia, In Kong L’s study, hrHPV plus methylation had similar positive predictive values (0.930 and 0.954, respectively, p = 0.395) for CIN2 detection, Additionally, hrHPV, methylation, and hrHPV plus methylation had similar negative predictive values (0.612, 0.679, and 0.655, p = 0.677) that were significantly higher than that of cytology alone (0.250, p values 0.012, 0.001, and 0.001, respectively), and for 49 cases with negative hrHPV results, positive methylation alone was able to differentiate CIN2+ from inflammation/CIN1 (11). Combining HPV typing with methylation detection may offer a more comprehensive and accurate approach for cervical cancer screening. By simultaneously detecting specific HPV types and the methylation status of related genes, it is possible to better identify women at high risk of developing cervical cancer, improve the sensitivity and specificity of screening, and reduce unnecessary referrals and overtreatment.

Unlike previous studies, this study focused on evaluating the diagnostic performance of hrHPV testing, DNA methylation, PAX1/ZNF671 status and the combination of hrHPV and PAX1/ZNF671 detection in paired urine samples, cervical samples and vaginal secretions for the diagnosis of high grade cervical intraepithelial neoplasia, exploring the optimizing cervical cancer screening strategies, and further assessed the diagnostic efficacy of PAX1 combined with ZNF671 in HPV-negative individuals.

## 2 Materials and methods

### 2.1 Study design and participants

This study was to detect the diagnostic effectiveness of different strategies from three -paired samples, and developed an assay of PAX1 and ZNF671 methylation for detection of CIN2+ and CIN3 +. The data regarding abnormal cytology and/or abnormal cervical biopsy pathology results for inpatients or outpatients of the Department of Obstetrics and Gynecology of the study center from April 2024 to September 2025 was performed. This study was approved by Ethics committee of The First Affiliated Hospital of Xinxiang Medical University(Approval EC-2024-656).

Based on the sample size calculated using SPSS, A total of 150 paired samples are planned for analysis; considering patients’ participation willingness and partial sample disqualification, we aim to enroll paired samples from 200 patients initially. Prior to enrollment, all patients provided written informed consent in accordance with the study protocol. The inclusion criteria were as follows: 1) Voluntarily participate in this study and sign the informed consent; 2) aged ≥25 years and ≤64 years; 3) Abnormal cervical cytology results (≥ASC-US) and/or abnormal cervical biopsy pathology results within 30 days before screening; 4) No contraindications to vaginal sampling and urine sample retention; 5) No history of cervical precancerous lesions, other malignant neoplasms, medical radiotherapy, organ transplantation, or immunosuppressive therapy; 6) HIV test results must also be negative. 7) No participation in other clinical studies within 30 days before screening. The exclusion criteria included the following: No current cytology test results within 30 days; Current treatment (LEEP, cervical conization, trachelectomy) for cervical lesions due to abnormal cervical cancer screening results before screening; Acute vaginal inflammation.

### 2.2 Screening method

For individuals who met the enrollment criteria, cervical scrapes and paired self-vaginal samples, urine-based samples should be conducted during the non-menstrual period. Individuals should avoid intravaginal medication administration, vaginal irrigation, sexual intercourse, and other related activities within 24 hours prior to sampling. The samples should be immediately placed into a special sample preservation solution and delivered to the laboratory for testing as soon as possible. The samples are subjected to hrHPV genotyping analysis as well as methylation analysis.

a complete urine void was collected using urine sample-specific collection tube followed by collecting vaginal secretions. Place the sampling brush inside the cervical canal, pressing it tightly around the external of the cervix. Rotate the brush slowly in one consistent direction for at least 5 full turns; Reverse rotation must be avoided. Cervex-Brush (Rovers Medical Devices, Oss, The Netherlands) was used to collect the clinician-taken cervical scrapes, and directly placed in Thinprep Preservcyt medium (Hologic, Marlborough, MA, US) and stored at 4°C.

### 2.3 Collection of study materials

This study adopted a double-blind design. After receiving the samples, the laboratory conducts the pretreatment of samples before detection in accordance with the following procedures: ① Record the sample numbers and complete the verification of sample types; ② Perform DNA extraction from the samples under aseptic operation; ③ Divide the extracted DNA samples into two equal parts on average—one part was used for HPV detection, and the other part undergone DNA conversion and purification for gene methylation detection.

For DNA extraction from cervical scrapes and vaginal self-samples, the Universal Nucleic Acid Extraction Kit (Magnetic Bead Method) produced by Cuizhen Biology (Zhengzhou) Co., Ltd. was adopted. The DNA isolation of urine utilized the Urine Nucleic Acid Extraction Kit (Magnetic Bead Method) manufactured by Cuizhen Biology (Zhengzhou) Co., Ltd. DNA Conversion and Purification utilized the Bisulfite Conversion and Purification Kit from Cuizhen Biology (Zhengzhou) Co., Ltd. Unconverted DNA can be stored at -20°C for 1 year and at -80°C for long-term storage. Converted DNA can be stored at 2°C–8°C for no more than seven days and at -20°C ± 5°C for no more than 6 months, with the number of freeze-thaw cycles not exceeding.

### 2.4 HPV DNA detection

All samples underwent HPV DNA analysis utilized a modified general primer-based polymerase chain reaction(PCR) assay, in accordance with the operating instructions of the PCR detection kit(Guangdong Kaipu Biotechnology Co., Ltd., China), HPV detection was performed using the PCR method, which included genotyping for HPV16/18 and qualitative detection of 12 other high-risk genotypes (HPV31, 33, 35, 39, 45, 51, 52, 56, 58, 59, 66, 68). The specific experimental procedures were as follows: thermal cycling parameters comprising an initial denaturation at 95°C for 3 minutes, 40 cycles of 95°C for 20 seconds, 58°C for 30 seconds, and 72°C for 45 seconds, followed by a final extension at 72°C for 7 minutes; and product purification via exonuclease I digestion to remove unincorporated primers prior to Luminex bead-based hybridization.

### 2.5 DNA methylation analysis

The promoter methylation status of the six gene(PAX1, ZNF671, JAM3, ZNF582, SOX11, and EPB) was analyzed using methylation-specific quantitative PCR (qMSP) based on TaqMan chemistry, the primers and probes used for the methylation detection of these genes are listed in **Supplementary Table 1**. According to the methylation results, PAX1 and ZNF671 were used as a representative gene for illustration. Methylation analysis was carried out with the commercial Methylated Human PAX1 and ZNF671 Gene Detection Kit (Zhengzhou Cuizhen Biotechnology Co., Ltd.China) on an ABI 7300 Real-Time PCR System (Thermo Fisher Scientific Co., Ltd.China).

Quantitative methylation specific PCR amplifications were performed according to the manufacturer’s instructions as described in previous literature (11).

A GAPDH-specific was served as an internal reference to confirm quantification and quality. The methylation level for each target gene was quantified using the ΔCt method, calculated as ΔCt_P_=Ct_PAX1_–Ct_GAPDH_ and ΔCt_Z_= Ct_ZNF671_-Ct_GAPDH_. If no amplification curve was observed for a target gene, its Ct value was manually set to 40. The sample was defined as methylation-“positive” if the ΔCt value for either PAX1 or ZNF671 was below its cutoff value. A sample was deemed “negative” only if both ΔCt_Z_ and ΔCt_P_ were above their respective cutoffs.

### 2.6 Statistical analysis

In this study, baseline data were analyzed based on the Full Analysis Set (FAS), which refers to all cases from which samples were collected; All efficacy indicators were analyzed using both the Full Analysis Set (FAS) and the Per-Protocol Set (PPS), the later refers to all cases that met the study protocol requirements, demonstrate good compliance, have completed sample collection and necessary colposcopic biopsy, and have fulfilled the content requirements for filling out the Case Report Form (CRF); and the Safety Analysis Set (SAS) was adopted for safety analysis.

All statistical analyses were performed using IBM SPSS Statistics, Version 30.0 (IBM Corporation, Armonk, USA). The optimal cutoff points for DNA methylation were determined using receiver operating characteristic (ROC) curve analysis and the Youden index (defined as specificity + sensitivity – 1), with reference to the distinction between cervical inflammation/CIN1 and CIN2 or more severe lesions. Non-normally distributed continuous variables and categorical data were compared across different screening groups using nonparametric tests. Additionally, the specificity, sensitivity, negative predictive value (NPV), and positive predictive value (PPV) were computed for each screening group. Negative Likelihood Ratio is defined as (1 - Clinical Sensitivity)/Clinical Specificity, Positive Likelihood Ratio defined as Clinical Sensitivity/(1 - Clinical Specificity). Unless specified otherwise, a two-sided significance level of 0.05 was adopted for all analyses.

## 3 Results

### 3.1 Patient characteristics

**Figure 1:**
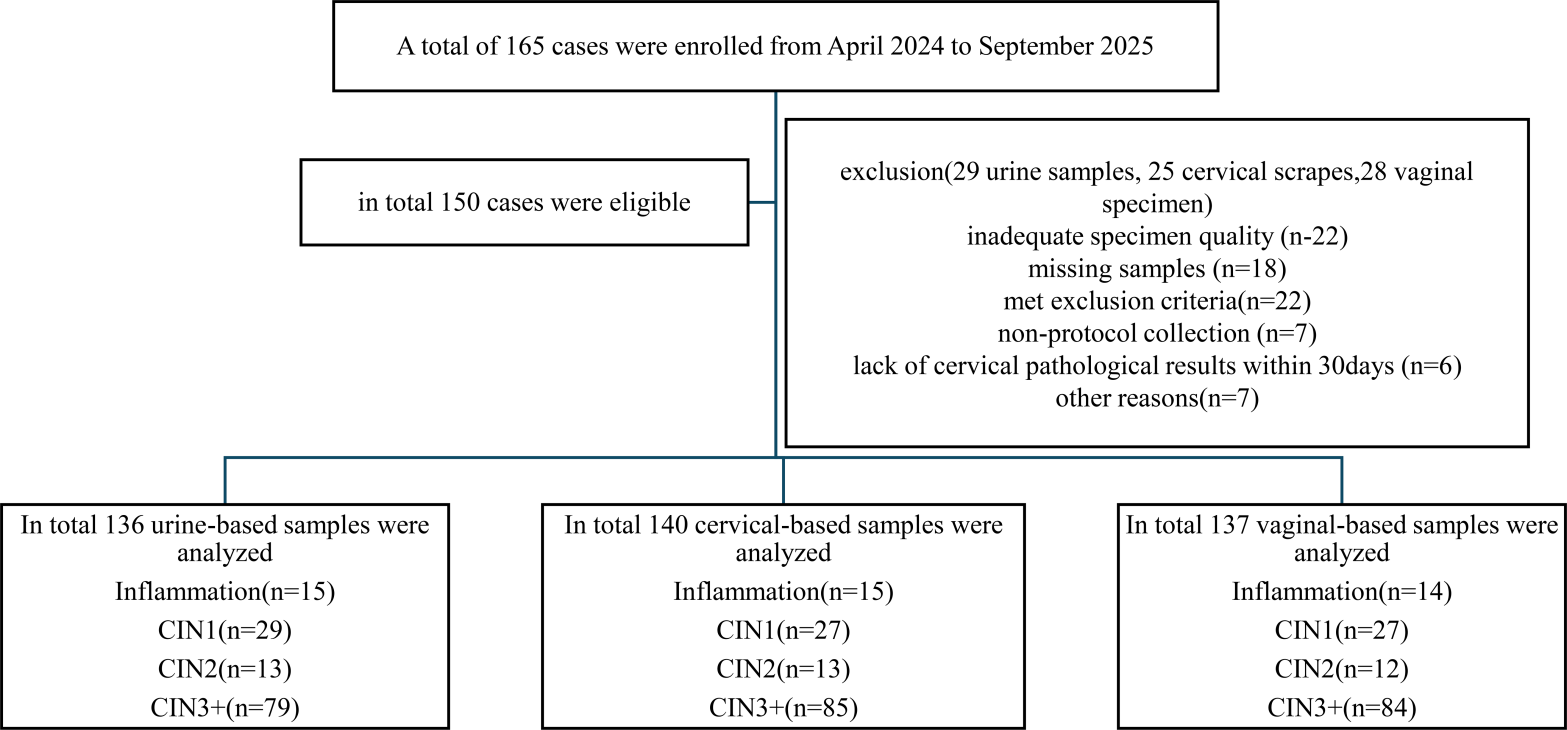
Flowchart of the study. CIN, cervical intraepithelial neoplasia.

In this study, study samples were collected from 195 women, and categorized into three groups based on specimen type: the urine-based sample group comprised 170 cases, of which 136 met the protocol requirements. A total of 34 cases were discontinued or excluded for various reason (e.g. unqualified specimens). 137 Vaginal samples and 140 clinic-taken cervical scrapes met the protocol criteria, the remaining were excluded for different reasons (e.g. incomplete sample-set, substandard quality, no recent cytological results) see **Figure 1**. The mean age of all participants was 44.82 years, with a range of 25 to 63 years. Based on the population’s previous pathological examination results, of 142 cases, 44(30.99%) was diagnosed as CIN1-, 12(8.45%) as CIN2, and 86(60.56%) as CIN3 +. The characteristics of study population was summarized in **Table 1**.

**Table 1:** Baseline characteristics of the study population.

Characteristic	Total	Urine	Cervical	Vaginal
Age	44.82(25;63)	45.27(25;63)	45.37(26;63)	44.70(25;63)
Cytology result at baseline visit, n (%)				
Negative	5(3.52%)	5(3.68%)	5(3.57%)	5(3.65%)
Positive	107(75.35%)	101(74.26%)	107(76.43%)	105(76.64%)
Unkown	30(21.13)	30(22.06%)	28(20.00%)	27(19.71%)
HPV test result at baseline visit, n (%)				
Negative	11(7.74%)	11(8.08.13%)	10(7.35%)	11(8.03%)
Positive	115(80.98%)	110(80.88%)	115(82.35%)	112(82.35%)
Unkown	15(10.56%)	15(11.03%)	15(10.71%)	14(10.29%)
Histopathological diagnosis, n (%)				
Normal or CIN1	44(30.99%)	44(32.35%)	42(30.15%)	42(30.66%)
CIN2	12(8.45%)	12(8.82%)	12(8.57%)	12(8.75%)
CIN3+	86(60.56%)	80(58.82%)	86(61.43%)	83(60.58%)

HPV positive is defined as testing positive for any of the following high-risk HPV types: HPV16, 18, 31, 33, 35, 39, 45, 51, 52, 56, 58, 59, 66, and 68. CIN, cervical intraepithelial neoplasia; CIN3+: lesions of CIN3 or more severe.

### 3.2 Cut off values of DNA methylation

**Figure 2:**
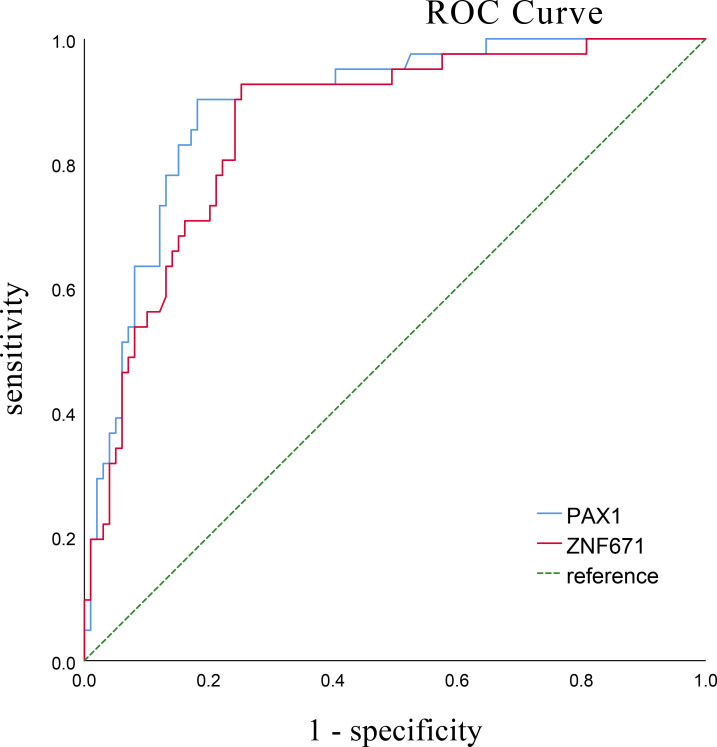
Receiver operating characteristic (ROC) curves for the performance of PAX1 and ZNF671, the methylation status of both gene by qMSP to differentiate cervical inflammation/cervical intraepithelial neoplasia (CIN) 1 from CIN2 or more severe cervical lesions. For PAX1 and ZNF671, the areas under the ROC curve were 0.846 and 0.892 (95% confidence interval 0.834 to 0.948 [p < 0.001] and 0.861 to 0.926 [p < 0.001]), respectively.

**Figure 3:**
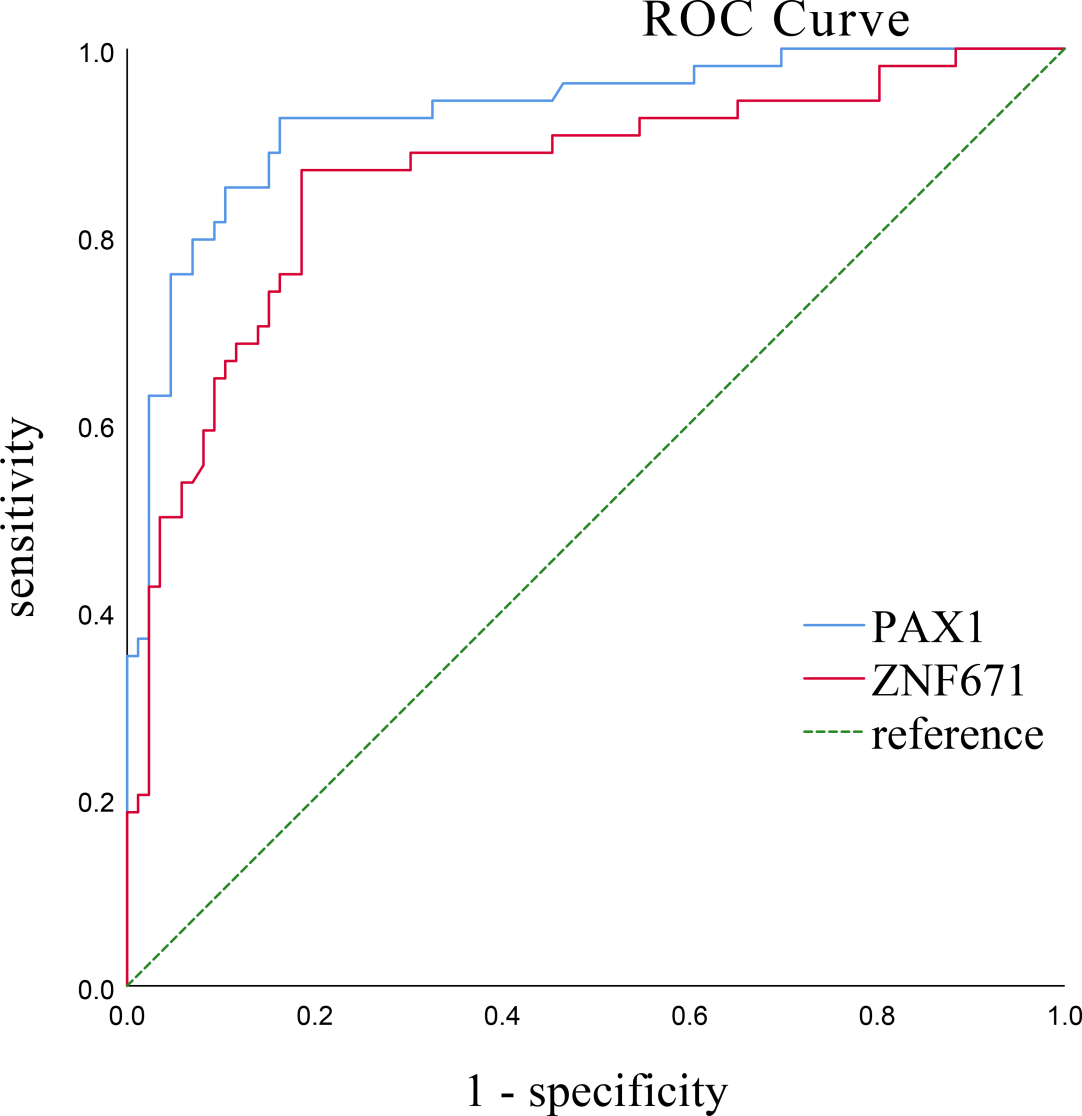
The ROC curves of PAX1 and ZNF671 methylation to differentiate cervical inflammation/cervical intraepithelial neoplasia (CIN) 1, CIN2 from CIN3 or more severe cervical lesions. For PAX1 and ZNF671, the areas under the ROC curve were 0.929 and 0.862 (95% confidence interval 0.884 to 0.974 [p < 0.001] and 0.795 to 0.929 [p < 0.001]), respectively.

The AUC of PAX1 and ZNF671 were analyzed and compared in cervical scrapes by receiver operating characteristic (ROC) curves across all 140 patients for detecting CIN2+ and CIN3 +. As shown in **Figures 2**, **3** For PAX1 and ZNF671, the areas under the ROC curve for CIN2+ were 0.846 and 0.892 (95% confidence interval 0.834 to 0.948 [p < 0.001] and 0.861 to 0.926 [p < 0.001]), respectively. The areas under the ROC curve of PAX1 and ZNF671 for CIN3+ were 0.929 and 0.862 (95% confidence interval 0.884 to 0.974 [p < 0.001] and 0.795 to 0.929 [p < 0.001]), respectively. The prevalence of methylation for both genes exhibited a strong positive correlation with the severity of cervical lesions (**Supplementary Tables S1**–**S3**). For PAX1, the methylation-positive rate increased from 12.19% (5/41) in CIN1- to 30.8% (4/13) in CIN2, and 89.5% (77/86) inCIN3+ lesions. A similar trend was observed for ZNF671, with methylation rates of 7.32% (3/41) in CIN1- to 30.8% (4/13) in CIN2, and 80.2% (69/86) in ≥CIN3.

Both genes showed high areas under the curve, based on sensitivity, specificity, and Youden index analyses, the cutoff values for PAX1 and ZNF671 methylation were 7.95and 10.92, respectively, with the highest Youden index values being 0.763 and 0.684, respectively.

### 3.3 Diagnostic accuracy of various screening methods across three paired samples for detecting CIN2+

As shown in **Table 2**, in Urine-based samples, hrHPV testing exhibited significantly higher sensitivity for CIN2+lesions compared to DNA methylation(92.39%% versus 55.43%, *P <*0.001) and PAX1/ZNF671 methylation (92.39%%versus 44.56%, *P <*0.001). No significant difference in sensitivity was observed between DNA methylation and PAX1/ZNF671 methylation (*P* = 0.074).

**Table 2:** The diagnostic accuracy of different screening methods for CIN2+ lesions across different samples.

Parameter	Urine		Cervical scrape			Vaginal		χ²/F	P
	Value	95%CI	Value	95%CI		Value	95%CI		
hr-HPV									
Sensitivity	92.39% (85/92)	85.47~96.45%	89.80% (88/98)	82.50~94.60%		92.63% (88/95)	85.98~96.64%	0.85	0.655
Specificity (CIN1-)	40.91% (18/44)	26.83~56.38%	42.8% (18/42)	28.57~58.21%		35.71% (15/42)	22.00~51.50%	0.53	0.645
PPV	76.58% (85/111)	68.15~83.51%	78.57% (88/112)	70.30~85.30%		76.52% (88/115)	68.20~83.40%	0.19	0.899
NPV	72.00% (18/25)	51.30~87.30%	64.29% (18/28)	44.70~80.60%		68.18% (15/22)	46.00~85.50%	0.58	0.659
PLR	1.56	1.19~2.05	1.57	1.20~2.06		1.44	1.10~1.88	–	0.910
NLR	0.19	0.09~0.40	0.24	0.12~0.48		0.21	0.10~0.44	–	0.629
PAX1/ZNF671									
Sensitivity	44.57% (41/92)	34.21~55.30%	85.8% (85/99)	77.54~91.74%		64.58% (62/96)	54.12~73.98%	21.38	<0.001
Specificity (CIN1-)	86.64% (39/44)	77.28~95.431%	85.3% (35/41)	73.15~93.10%		82.93% (34/41)	69.15~91.855	1.15	0.563
PPV	89.13%(41/46)	78.25~95.64%	93.4% (85/91)	86.54~97.23%		89.86% (62/69)	80.75~95.61%	2.12	0.346
NPV	43.33% (39/90)	33.25~53.78%	71.4% (35/49)	57.74~82.60%		50.00% (34/68)	38.24~61.76%	7.96	**0.019**
PLR	3.92	1.83~8.41	5.80	2.65~12.68		3.78	1.85~7.72%	1.89	0.389
NLR	0.62	0.51~0.76	0.17	0.10-0.29		0.43	0.32~0.58	18.75	**<0.001**
DNA methylation									
Sensitivity	55.43% (51/92)	44.89~65.52%	89.8% (88/98)	82.21~94.68%		71.88% (69/96)	61.78~80.42%	34.52	**0.000**
Specificity	81.82% (36/44)	67.34~91.26%	57.1% (24/42)	41.52~71.73%		53.66% (22/41)	37.89~69.01%	7.58	0.031
PPV	86.44% (51/59)	75.01~93.58%	83.0% (88/10)	75.01~89.12%		78.41% (69/88)	68.98~85.83%	2.87	0.238
NPV	46.75%(36/77)	35.61%~58.10%	70.59% (24/34)	53.52~83.87%		44.90% (22/49)	31.28~59.12%	12.45	**0.002**
PLR	3.04	1.68~5.50	2.05	1.43~2.94		1.55	1.06~2.27	21.78	**0.000**
NLR	0.54	0.42~0.70	0.18	0.10~0.32		0.52	0.38~0.72	25.31	**0.000**

CIN1-: Normal, inflammation, or CIN grade 1; CIN2+: CIN grade 2 or higher lesions. Data represent no. (%) of study participants unless otherwise specified. hrHPV: HPV16, 18, 31, 33, 35, 39, 45, 51, 52, 56, 58, 59, 66 and 68. Methylation detection was performed using the Methylation-Specific PCR (MSP) method. Samples with positive MSP results were recorded as methylation-positive, and others as methylation-negative. PAX1/ZNF671 positive defined as positive either or both for PAX1 and ZNF671. methylation positive is defined as at least one of the six genes (PAX1, ZNF671, JAM3, ZNF582, SOX11, and EPB gene) showing positive for methylation. Negatvie is defined as all six genes tested negative for methylation.

n, number; CIN, cervical intraepithelial neoplasia; HPV, human papillomavirus; hrHPV, high-risk HPV; PPV, Positive Predictive Value; NPV, Negative Predictive Value; PLR, Positive Likelihood Ratio; NLR, Negative Likelihood Ratio.

Bold values indicate statistically significant differences in the diagnostic accuracy of different screening methods for CIN2+ among different sample types.

The specificity of both methylation assay were significantly higher than that of hrHPV testing for CIN2 lesions (all P <0.001), and this trend also holds true for the comparison of Positive Predictive Value(PPV)(*P* = 0.003, 0.038 respectively), which exhibits a significantly higher triage efficacy. The NPVs of PAX1/ZNF671 and DNA methylation assay were all significantly lower than that of hrHPV (46.75%, 43.33% vs 72.00%).

For cervical scrape samples, hrHPV testing, DNA methylation and PAX1/ZNF671 methylation had comparable sensitivity for CIN2+ (89.80%, 85.86%, 89.80%; *P* = 0.170), while hrHPV and DNA methylation showed similar specificity (42.86% vs 57.14%, *P* = 0.150) — both significantly lower than PAX1/ZNF671 methylation (85.37%, *P* < 0.001 and *P* = 0.001, respectively). Correspondingly, PAX1/ZNF671 methylation achieved the highest PPV (93.41%, 95%CI:87.00~97.10%) and strongest Positive Likelihood Ratio(PLR) (5.80, 95%CI:2.65-12.68) for CIN2 +. NPV was comparable across the three methods (70.59%, 71.43%, 64.29%; *P* = 0.306), and broader DNA methylation and PAX1/ZNF671 methylation had similar NLR (0.17 vs 0.18), both lower than hrHPV testing (0.24), indicating superior discriminatory ability for CIN2 +.

Consistent with urine samples, hrHPV testing had significantly higher sensitivity for CIN2+ than DNA methylation and PAX1/ZNF671 methylation ((92.63% vs 71.88% and 64.58%, all *P* < 0.001)), with no sensitivity difference between the two methylation assays. PAX1/ZNF671 methylation had the highest specificity (82.93%), significantly higher than hrHPV (35.71%) and DNA methylation (53.66%, *P* < 0.001), and correspondingly yielded the highest PPV (89.86% vs 76.52%, 78.41%, *P* = 0.013) and PLR (3.78 vs 1.44, 1.55, *P* < 0.001). hrHPV testing had the lowest NLR (0.21 vs 0.43, 0.52, *P* < 0.001), and NPV was comparable across all three methods.

### 3.4 Diagnostic performance of hrHPV testing for the differentiate of CIN2+ and CIN3+ from different samples

For hrHPV testing, CIN2+ detection across cervical scrape, vaginal and urine samples showed equally high sensitivity (89.80% to 92.63%, *P* = 0.655) and low, comparable specificity (35.71% to 42.80%, χ²=0.53, *P* = 0.645); PPV, NPV, PLR and NLR also did not differ significantly among the three sample types (all *P*>0.05), indicating equivalent diagnostic efficacy for CIN2 +.Concerning CIN3+ detection, hrHPV testing exhibited exceptionally high, statistically equivalent sensitivity across the three sample types (93.75% urine, 94.12% cervical scrape, 93.98% vaginal; *P* = 0.994), with no significant intergroup differences in specificity, PPV or NPV (**Table 3**).

**Table 3:** The diagnostic accuracy of different screening methods for CIN3+ lesions across different samples.

Parameter	Urine		Cervical scrape		Vaginal		χ²/F	*P*
	Value	95%CI	Value	95%CI	Value	95%CI		
HPV								
Sensitivity	93.75% (75/80)	88.83%~98.67%	94.12% (80/85)	87.57%~97.91%	93.98% (78/83)	87.00%~97.90%	0.012	0.994
Specificity (CIN2-)	35.71% (20/56)	22.84%~48.58%	41.82 (23/55)	28.68%~55.83%	31.48% (17/54)	20.10~45.40%	0.876	0.645
PPV	67.57% (75/111)	58.95%~76.19%	71.43% (80/112)	62.41%~79.38%	67.83% (78/115)	58.60~76.10%	0.589	0.745
NPV	80.00%(20/25)	65.22%~94.78%	82.14% (23/28)	64.35%~93.09%	77.27% (17/22)	55.80%~91.40%	0.198	0.906
PLR	1.458	1.16-1.81	1.618	1.153~2.275	1.37	1.15~1.64	4.328	**0.015**
NLR	0.175	0.08-0.40	0.141	0.058~0.342	0.19	0.08~0.47	0.356	0.702
PAX1/ZNF671 methylation								
Sensitivity	48.10% (38/79)	36.82%~59.54%	91.86% (79/86)	84.05%~96.64%	71.43% (60/84)	61.0%~80.3%	45.927	**<0.001**
Specificity	85.96% (49/57)	75.09%~93.27%	77.78% (42/54)	65.09~87.27	83.02% (44/53)	72.1%~90.7%	3.865	0.145
PPV	82.61% (38/46)	69.23%~91.45%	86.81% (79/91)	78.34%~92.74%	86.96% (60/69)	77.6%~93.2%	0.712	0.699
NPV	54.44% (49/90)	43.78%~64.85%	85.71% (42/49)	72.73%~93.70%	64.71% (44/68)	53.5%~74.8%	18.753	**<0.001**
PLR	3.44	2.05~5.77	4.13	2.45 ~ 6.96	4.21	2.36~7.43	4.892	**0.010**
NLR	0.60	0.49~0.74	0.10	0.04 ~ 0.23	0.34	0.24~0.48	31.268	**<0.001**
DNA methylation								
Sensitivity	60.76% (48/79)	49.23%~71.38%	94.12% (80/85)	87.57%~97.91%	76.19% (64/84)	66.2%~84.2%	38.652	**<0.001**
Specificity	80.70% (46/57)	69.01%~89.27%	52.73% (29/55)	39.10%~66.07%	54.72% (29/53)	42.6%~66.3%	14.587	**0.001**
PPV	81.36% (48/59)	69.45%~89.82%	75.47% (80/106)	66.58%~82.93%	72.73% (64/88)	62.9%~80.9%	2.134	0.344
NPV	59.74% (46/77)	48.03%~70.61%	85.29% (29/34)	69.84%~94.08%	59.18% (29/49)	46.6%~70.8%	10.269	**0.006**
PLR	3.15	1.87~5.32	1.99	1.41~2.81	1.68	1.23~2.29	27.891	**<0.001**
NLR	0.49	0.38~0.62	0.11	0.04~0.26	0.43	0.29~0.63	18.765	**<0.001**

Bold values indicate statistically significant differences in the diagnostic accuracy of different screening methods for CIN3+ among different sample types.

### 3.5 The diagnostic performance of DNA methylation testing for detecting CIN2+ and CIN3+ lesions

The capacity of the individual markers (PAX1, ZNF671, JAM3, ZNF582, SOX11, and EPB) to differentiate high grade lesions (CIN2+ and CIN3+) was compared in three paired samples types (**Tables 2**, **3**). Sensitivity for CIN2+ and CIN3+ was highest in cervical scrapes (89.8%,95%CI:82.21~94.68%; 94.12%, 95%CI: 87.57% ~ 97.91%), intermediate in vaginal samples (71.9%,76.2%), and lowest in urine (55.4%,60.8%) (all P<0.001). In contrast, specificity exhibited an opposite trend to sensitivity across the three sample types(P<0.05). The capacity to exclude CIN2+ and CIN3+ diseases was strongest for cervical scrapes, as evidenced by their significantly higher NPV (85.3% and 70.6%, P = 0.006,0.002 respectively), and superior NLR (all P<0.001).

### 3.6 Value of PAX1/ZNF671 methylation assay across sample types for high grade CIN detection

Through methylation testing, we found PAX1 and ZNF671 had higher Sensitivity and specificity(see **Supplementary Tables S2**–**S4**), therefore, the diagnostic value of PAX1/ZNF671 methylation was further evaluated. The results were summarized in **Tables 2**, **3**, The diagnostic performance of the PAX1/ZNF671 methylation assay was evaluated and compared for the detection of high-grade CIN. The findings demonstrated a relative correlation of diagnostic performance and sample types.

For CIN2+ Detection, the aforementioned indices were calculated for all conditions reported above samples, a higher sensitivity was observed in cervical scrapes (85.86%, 95%CI:77.54-91.74%) than vaginal (64.58%, 95%CI:54.12-73.98%) and urine (44.57%, 95%CI: 34.21-55.30%) samples (*P* < 0.001). An equivalent high specificity and PPV were observed across three sample types. The cervical scrape had the highest NPV (71.4% vs 43.33%, 50.00%, *P* = 0.019) and the lowest NLR (0.17 vs 0.62, 0.43 *P* < 0.001) among three sample types.

Concerning CIN3+ detection, cervical scrape researched the highest diagnostic sensitivity than that of the other two sample types (91.86% vs 48.10%, 71.43%, *P* < 0.001). While the specificity was all above 77.78% (*P* = 0.145), the NPV for cervical scrapes (85.71%) was substantially and significantly higher than that of the self-collected alternatives (*P* < 0.001). The diagnostic power was also strongest for cervical scrapes (NLR = 0.10), presenting a pronounced advantage over vaginal and urine samples (*P* < 0.001).

### 3.7 The diagnostic value of PAX1/ZNF671 in HPV negative population

**Supplementary Tables S5**, **S6** showed the diagnostic performance of PAX1/ZNF671 in HPV negative population, whether CIN2+ or CIN3+, cervical scrapes had a significant higher sensitivity (80%,100%) than the other self-collected specimen, there were significant statistical difference (χ²=24.89, 16.83, all *P* < 0.001).

### 3.8 Comparative diagnostic accuracy of hrHPV and DNA methylation across sample types for CIN2+ and CIN3+ detection

The results summarized in **Tables 4**, **5**, an obvious finding was the consistently superior sensitivity of cervical scrapes for detecting both CIN2+ and CIN3 +. For CIN3+ lesions, the sensitivity of cervical scrapes was 96.51%(95%CI: 90.17-99.08%), significantly higher than that of vaginal (82.35%,95%CI: 72.63-89.54%) and urine (73.75%, 95%CI: 62.98-82.45%) samples (*P* = 0.0002). This trend was equally pronounced for CIN2+ lesions, where cervical scrapes achieved a sensitivity of 94.90%, compared to 80.39% for vaginal and 74.00% for urine samples (*P* < 0.001). Which established cervical scrapes as the most reliable sample for distinguishing high-grade cervical lesions. For both CIN2+ and CIN3+, the NPV of cervical scrapes were significantly higher (83.33% and 90.00%, respectively) than that of vaginal and urine samples (*P* = 0.002,0.0049 respectively). Concordantly, the NLR for cervical scrapes was exceptionally low (0.09 for CIN2+, 0.07 for CIN3+), significantly outperforming the other sample types (*P=*0.000, 0.0007). In contrast to the significant differences in sensitivity and NPV, the specificity of the assay was similar low across all sample types (48% - 59% for CIN2+, 48%-50% for CIN3+), with no statistically significant differences observed (all *P*> 0.05). The three samples had similar moderately high PPV for CIN2+ and CIN3+(all *P* < 0.05).

**Table 4:** The diagnostic accuracy of hrHPV in combination with DNA methylation for CIN2+ lesions from different samples.

Parameter	Urine		Cervical scrape		Vaginal		χ²/F	*P*
	Value	95%CI	Value	95%CI	Value	95%CI		
Sensitivity	74% (67/90)	61.84~ 80.28%	94.90% (93/98)	88.17~ 97.43%	80.39% (80/102)	71.78~ 87.13%	19.876	**0.000**
Specificity	53.49% (23/43)	38.65~ 68.33%	59.52% (25/42)	38.17~ 69.03%	57.14% (20/35)	40.13~ 72.95%	0.782	0.676
PPV	77.01% (67/87)	(68.24~ 85.78%	84.55% (93/110)	77.31~ 90.04%	84.54% (82/97)	76.47~ 90.63%	3.158	0.207
NPV	46.94% (23/49)	32.98~ 60.90%	83.33% (25/30)	66.04~ 93.47%	50.00% (20/40)	34.52~ 65.48%	12.453	**0.002**
PLR	1.53	1.12~2.15	2.34	1.62~3.38	1.87	1.33~2.63	/	**0.012**
NLR	0.54	0.37~0.73	0.09	0.06~0.12	0.35	0.22~0.57	/	**0.000**

CIN2+, cervical intraepithelial neoplasia grade 2 or worse; hrHPV, high-risk human papillomavirus; PPV, positive predictive value; NPV, negative predictive value; PLR, positive likelihood ratio; NLR, negative likelihood ratio; CI, confidence interval.

Bold values indicate statistically significant differences among the three samples in distinguishing CIN2 (P<0.01).

**Table 5:** The diagnostic accuracy of hrHPV combined with DNA methylation for CIN3+ lesions among different samples.

Parameter	Urine		Cervical scrape		Vaginal		χ²/F	*P*
	Value	95%CI	Value	95%CI	Value	95%CI		
Sensitivity	73.75% (59/80)	62.98~82.45%	96.51% (83/86)	90.17~99.08%	82.35% (70/85)	72.63~89.54%	16.89	**0.0002**
Specificity	50.00% (28/56)	36.65~63.35%	50.00% (27/54)	36.30~63.70%	48.08% (25/52)	34.58~61.83%	0.08	0.9605
PPV	67.82% (59/87)	57.21~76.98%	75.45% (83/110)	66.42~83.05%	72.16% (70/97)	62.23~80.53%	2.53	0.2827
NPV	57.14%(28/49)	42.68~70.73%	90.00% (27/30)	74.48~97.23%	62.50% (25/40)	46.23~76.47%	10.65	**0.0049**
PLR	1.48	1.09~2.00	1.93	1.41~2.65	1.59	1.16~2.15	3.12	0.2101
NLR	0.53	0.36~0.78	0.07	0.02~0.20	0.37	0.24~0.57	14.73	**0.0007**

CIN3+, cervical intraepithelial neoplasia grade 3 or worse.

Bold values indicate statistically significant differences among the three samples in distinguishing CIN3+(P<0.01).

## 4 Discussion

As an effective strategy for the secondary prevention of cervical cancer, comprehensive and highly effective screening was critical to the global goal of eliminating the disease. Self-sampling for HPV detection was a validated, non-inferior alternative to clinician-based collection, crucial for expanding cervical cancer screening in resource-poor settings. Evidence confirmed its comparable sensitivity(0.98 [95% CI 0.95-1.01]) and slightly more specific (1.03 [1.02-1.04]) for CIN2+ (12). Notably, the safety and effective from different samples should be invective.

The primary objective of this study was to comprehensively evaluate and compare the diagnostic performance of three distinct molecular methods —hrHPV testing, PAX1/ZNF67 gene methylation, and DNA methylation assay—for the detection of CIN2+ and CIN3+ lesions, utilizing three paired self-collected and clinician-collected sample types. The principal finding of this research were as follows: For hrHPV testing, self-collected urine and vaginal samples demonstrated non-inferior diagnostic value for the detection of both CIN2+ and CIN3+ lesions compared to clinician-collected cervical scrapes. Statistical analysis confirmed a highly significant association between the methylation status of PAX1/ZNF67 and histopathological grade. Another, the performance of methylation-based assays was profoundly influenced by the source of the sample, with cervical scrapes consistently yielding superior diagnostic accuracy. Most importantly, PAX1/ZNF671 methylation testing exhibited favorable diagnostic efficacy in HPV-negative populations.

Unlike cytology testing, which was more likely to be adversely affected by self-sampling, molecular testing for HPV DNA or RNA are less affected by specimen adequacy. Many studies demonstrated that HPV self-sampling has been shown to be feasible and acceptable in cervical cancer screening, and is a viable approach to screening population (13). In our cohort, the consistent high sensitivity of hrHPV testing observed in our study, exceeding 89% across urine, cervical, and vaginal samples, further demonstrate its well-established role as a highly effective primary screening tool (14, 15). In agreement with our findings, Song J et al. reported that the sensitivity shows a high consistency between cervical cytology and urine sample testing, with a kappa value of 0.637 (*P* < 0.01), and concluded that urine human papillomavirus testing, as a non-invasive screening method, has significant application effects (16). In another study, also demonstrated the same findings by showing that urine has a good concordance to the vaginal (k = 0.66) self-samples and cervical samples (k = 0.66) for hrHPV detection (17). These findings are particularly significant for the potential expansion of cervical cancer screening coverage. The comparable sensitivity achieved using self-collected urine and vaginal samples suggests that these methods could effectively overcome significant barriers to screening, such as cultural hesitancy, logistical challenges, and limited healthcare access, thereby reaching underserved populations (18).

However, the persistently low specificity of hrHPV testing, which ranged from only 35.71% to 42.8% in our cohort, remained its principal limitation. This high false-positive rate lead to substantial over-referral for colposcopy, resulting in patient anxiety, increased healthcare costs, and potential overtreatment of lesions that might otherwise regress (19). Our data confirmed that while hr-HPV was an excellent test for excluding disease (high Negative Predictive Value), its utility in definitively identifying which hrHPV positive women harbor significant histopathological lesions was limited, irrespective of the sample type, consequently, more accurate methods for cervical cancer screening strategies are required.

As high-risk human papillomavirus (hrHPV) testing—a highly sensitive yet moderately specific assay—becomes the gold standard for cervical screening, the development of effective triage strategies becomes imperative. Recently, DNA methylation biomarkers have been studied as triage tools for hrHPV-positive individuals to reduce unnecessary colposcopy referrals and prevent over-diagnosis and over-treatment (20, 21). Previously, host gene methylation might constitute a useful referral triage tool of hr-HPV+ women enrolled in the Cervical Cancer Screening Program to mitigate over-diagnosis and over-treatment, thereby addressing a critical need in ASC-US triage (22). However, their clinical translation required further validation through robust studies. A study indicated that the methylation assay demonstrated a sensitivity of 83.8% and a specificity of 95.8%, outperforming HPV-DNA testing in differentiating high-grade cervical lesions among women with ASC-US. Moreover, PAX1^m^/JAM3^m^ testing significantly reduced the colposcopy referral rate for further diagnostic procedures in high-risk HPV-positive women by 79.5% (23). As reported by Wen Y et al. in 2022, the PAX1/ZNF582 methylation detection has the highest specificity(97.30%) and accuracy, the HPV typing quantitative detection has the highest sensitivity(89.71%), but poor specificity (24), which was consistent with our results, namely, the DNA methylation triage testing which include PAX1, ZNF671, JAM3, ZNF582, SOX11 and EPB for CIN2 + was further investigated among ASC-US cases from three specimens, Sensitivity was highest for cervical scrapes(89.8%, 82.21~94.68%), intermediate for vaginal samples (71.88%, 61.78~80.42%), and lowest for urine (55.43%, 44.89~65.52%), which may be attributed to the detection methods and urine storage conditions (25), As reported by Burdier FR et al (26), In cervical cancer screening, urine-based methylation tests typically show lower sensitivity compared to cervical samples due to dilution of cervical cells in urine and additional DNA degradation during passage through the urinary tract. Subsequent research will further optimize the experimental conditions and explore the diagnostic efficacy of methylation levels in urine samples for CIN3 +. Moreover, methylation status has a better specificity, PPV, PLR and NLR to the hr-HPV testing from three samples, which indicated that positive methylation status showed a significantly stronger indicative effect for CIN2+ and CIN3+ than the hrHPV test. And a negative methylation results essentially exclude a diagnosis of CIN2+, especially CIN3 +. Therefore, methylation status can serve as an auxiliary screening indicator for CIN2+ in the ASC-US population. DNA methylation had a good discrimination in the ASC-US population. The triage efficiency of DNA methylation in our study was in accordance with previous reports (11, 27, 28).

Previous research had demonstrated that the ZNF671 methylation (ZNF671m) assay outperformed other molecular triage tests, including HPV16/18 genotyping and PAX1m, for detecting CIN3+ (29, 30). And further revealed that using ZNF671m as a triage strategy for hrHPV-positive women achieved comparable sensitivity(79.46%) but higher specificity(79.88%) than Thinprep cytologic test (TCT) (31). Another study demonstrated that the methylation of ZNF671 is measurable in cervical FFPE material and has prognostic value (32). As reported in some studies, the methylation detection of the PAX1 gene exhibited a higher diagnostic performance and may be a promising method for cervical cancer screening (33–35). Building on previous research, our study further validates the diagnostic performance of PAX1/ZNF671 in cervical cancer screening. However, our results revealed that this potential was not uniformly realized across all sample types. The PAX1/ZNF671 methylation assay exhibited exceptional performance on cervical scrape samples, with a sensitivity of 85.8% and a specificity of 85.3% for CIN2+, 91.86% and 77.78% for CIN3+, resulting in a remarkably high PPV of 93.4% and 86.81% respectively for CIN2+ and CIN3 +. This positions it as an nearly ideal triage test for hrHPV-positive women. By applying this test to a cervical sample, a significant proportion of women with transient, non-progressive HPV infections could be identified and safely returned to routine surveillance, thereby drastically reducing unnecessary colposcopy referrals without compromising sensitivity for detecting CIN2+ and CIN3+ (36).

Our analysis revealed that the combination of hr-HPV testing and DNA methylation analysis in cervical scrapes demonstrated high diagnostic performance, particularly with respect to sensitivity and negative predictive value (NPV). For the endpoints of CIN2+ and CIN3+, sensitivity values exceeded 94%, while NPV reached 83.33% for CIN2+ and 90.00% for CIN3+, which indicated that a negative test outcome reliably rule out the presence of a clinically significant cervical lesion. This conclusion was further supported by the low negative likelihood ratios (NLRs) observed—0.09 for CIN2+ and 0.07 for CIN3+—underscoring the strength of the combined assay in ruling out disease. Coincidentally, Lin C et al. reported that the diagnostic accuracy of combining HPV16/18 testing with all candidate gene methylation tests for the diagnosis of CIN2 + was significantly greater than when HPV16/18 testing was combined with cytology (37). This combined approach had an AUC of 0.907(95% CI: 0.858-0.955), a sensitivity of 72.73%(95% CI: 0.619-0.814), and a specificity of 98.58%(95% CI: 0.964-0.995), and proposed that combined HPV16/18 and multigene methylation testing for the triage of CIN2 + were significantly better than combined HPV16/18 and cytology testing (37). These findings have been corroborated by other studies, which consistently reported that the integrated assessment of HPV status and methylation markers possesses significant discriminatory power for CIN2+ lesions (38). A negative integrated result served to exclude the presence of CIN2+ (11, 39).

In agreement with the study by Cho HW et al. (40), who reported that relative sensitivity of realtime HR-S and Anyplex HPV tests for the detection of CIN2+ in vaginal versus cervical samples were 0.91 (95% CI, 0.90 to 1.03) and 0.87 (95% CI, 0.75 to 1.02), respectively, urine versus cervical comparisons were 0.79 (95% CI, 0.70 to 0.92) and 0.74 (95% CI, 0.61 to 0.89), and concluded that the detection performance for hrHPV and CIN2+ on self-collected vaginal samples was comparable to that of clinician-collected cervical samples. On the other hand, HPV tests using urine were inferior to those using clinician-collected cervical samples in terms of detecting hrHPV and CIN2 +. We observed that the self-collected samples of hrHPV testing from urine and vagina, showed a similar sensitivity for detecting CIN3 or more invasive diseases with the cervical scrapes (93.75%, 93.98% and 94.12%). Snoek BC et al (41) also reported that methylation levels in urine were moderately to strongly correlated to those detected in cervical scrapes (r = 0.508- 0.717). All DNA biomarkers were significantly increased in urine from cervical cancer patients compared to controls and showed a good discriminatory power for cervical cancer (AUC = 0.744-0.887).

Our results were consistent with those of van den Helder R et al (28), who found that DNA methylation levels strongly correlated with cervical lesion severity, achieving an AUC of 0.84 for CIN3+ detection (sensitivity: 86%; specificity: 70%), and concluded that hrHPV and methylation testing in urine was a highly promising strategy for detecting cervical neoplasia, especially for underscreened populations. Moreover, cervical samples demonstrated the lowest Negative Likelihood Ratio (NLR) (0.09 for CIN2+, 0.07 for CIN3+), indicating that they have the lowest likelihood of missed diagnoses in cervical cancer screening compared to self-collected urine and vaginal samples.

Our data provided compelling evidence for the efficacy of DNA methylation as an independent screening method for CIN2 +. This conclusion was consistent with previous studies that have reported strong clinical performance for other DNA methylation biomarkers in identifying high-grade cervical lesions (42, 43). As Schreiberhuber L et al. reported in 2024, a combination of WID-qCIN (assess the DNA methylation of human genes DPP6, RALYL and GSX1) and HPV16 and/or HPV18 (HPV16/18) reached a success rate of 93.4% in detecting cervical intraepithelial neoplasia grade 3 and 100% of invasive cervical cancers. The WID-qCIN/HPV16/18 combination predicted 69.4% of incident cervical intraepithelial neoplasia grade 2 or worse compared with 18.2% predicted by cytology (42). In this study, the sensitivity, PPV, NPV, and PLR of DNA methylation in cervical sample for detecting CIN2+ or CIN3+ were consistently high with that reported in the literature (44–46), further supporting its utility as an independent triage strategy for women with abnormal cytology result or hrHPV infection.

Based on the comprehensive diagnostic accuracy data presented in **Table 5**, cervical scrapes represent the most suitable sample type for cervical cancer screening via DNA methylation analysis, outperforming both urine and self-collected vaginal samples. The primary rationale for this conclusion rests on the superior performance profile of cervical scrapes. They demonstrated exceptional sensitivity (94.12%) for detecting CIN3+ lesions, which was paramount for a screening test to minimize false negatives. This was coupled with a remarkably low negative likelihood ratio (NLR = 0.11), indicating an excellent distinguished capacity. Therefore, when diagnostic accuracy was the primary consideration, clinician-collected cervical scrapes are preferred method for DNA methylation screening. Which was consistent with the Sellors JW et al’s study (47).

The comprehensive diagnostic efficacy of vaginal samples methylation testing for cervical diseases was inferior to that of cervical samples, with a 76.19% of sensitivity and slightly higher NLR (0.43) render them less perfect for primary screening, however, as an easy and non-invasive screening strategy, Vaginal self-sampling represented a viable alternative for individuals who were unable to access clinician-collected cervical sampling. This approach offers a practical strategy for expanding cervical cancer screening coverage in resource-limited settings (48, 49). For combination of hrHPV and methylation, urine samples had a poor diagnostic accuracy than reported in previous study (28, 50). Otherwise, For hr-HPV testing, the above methods have similar diagnostic efficacy, urine-based testing, the sensitivity was higher than that of previous study reported, wing to its ease of collection and non-invasiveness, offers a highly acceptable alternative that could overcome key barriers to attendance in cervical screening and provides a well-accepted screening option (16, 17).

Our study should be strengthened, firstly, six DNA methylation(PAX1, ZNF671, JAM3, ZNF582, SOX11 and EPB) analysis were performed from three paired samples simultaneously, and demonstrated that DNA methylation particularly PAX1/ZNF671 had a favorable differentiated from CIN2+,especially CIN3+; As underscreened populations (e.g., rural women in cervical cancer screening) still face challenges in early detection (51), the methylation detection method developed in this study, after optimizing for sample type-specific characteristics, can provide a more accurate, convenient, and non-invasive detection tool for clinical practice. Secondly, among the three paired samples, cervical samples were the perfect candidates for DNA methylation and combination testing, self-collected urine and vaginal samples had a moderately accepted triage capacity of DNA methylation combination testing, while self-urine based and vaginal samples had an equal diagnostic performance with cervical scrapes for hrHPV testing; Subsequently, we found the methylation testing of PAX1 and ZNF671 genes demonstrates optimal sensitivity and specificity, and their combined detection significantly have a better diagnostic value, which represents a novel perspective that was absent from the existing literature. Clarifying the performance differences across sample types can help clinicians select appropriate sample types based on specific clinical scenarios, optimize detection strategies, and improve the accuracy of early diagnosis and risk stratification of diseases. Another highlight of this study was that PAX1/ZNF671 methylation assays have a strong capacity in screening and excluding≥CIN2 lesions among HPV-negative individuals. In the long run, this study provides a theoretical and experimental basis for the clinical popularization of gene methylation detection technology, which is expected to promote the transformation of precision medicine from basic research to clinical application (52), reduce the mortality rate of related diseases through early screening and accurate diagnosis, and bring substantial clinical benefits to patients — aligning with the potential of blood-based multi-cancer early detection tests to alleviate cancer burden by addressing gaps in current screening programs (51).

However, certain limitations should be acknowledged. Firstly, this study is limited by a small sample size and its single-center design, a larger study population was expected to evaluate the strategy capacity of self-collected samples in future. Furthermore, the generalized ability of our findings may depend on the specific DNA methylation markers used in our assay. Another, this study did not distinguish between HPV vaccinated population and the unvaccinated population, future studies should take the HPV vaccination status into account to evaluate the efficacy of different screening method from different samples.

In conclusion, our study provides a critical evaluation of modern molecular diagnostics for cervical precancer. We confirmed that hrHPV testing was a robust primary screening method whose sensitivity was resilient to sample type, but the low specificity warrants an effective triage strategy. We demonstrate that PAX1/ZNF671 and individual DNA methylation assays represent highly promising triage tools, but their efficacy was exquisitely dependent on the sample types, cervical scrapes remained the optimal sample for methylation triage, achieving a balance of high sensitivity and specificity that could significantly improve the efficiency of cervical cancer screening programs.

Future efforts will focus on optimizing methylation detection techniques for self-collected samples, specifically, we will improve the extraction efficiency and purity of methylated DNA from self-collected samples, optimize the reaction conditions of methylation detection assays (such as PCR amplification parameters, probe design, etc.), and reduce the interference of impurities in self-collected samples on detection results, so as to improve the stability, sensitivity and specificity of methylation detection for self-collected samples. In addition, combined with the limitations of small sample size and single-center design in this study, future research will also expand the sample size and carry out multi-center studies to enhance the representativeness of the research subjects and the generalizability of the research conclusions.

## Data Availability

The original contributions presented in the study are included in the article/**Supplementary Material**. Further inquiries can be directed to the corresponding authors.
