# Supplementary material for: Comparative performance of hrHPV testing and PAX1/ZNF671 methylation in triaging women with abnormal cytology: a study of paired urine, vaginal and cervical scrape samples
Source: Front Oncol. 2026 Mar 11;16:1765689. doi: 10.3389/fonc.2026.1765689 (PMC13013482; doi:10.3389/fonc.2026.1765689)
Supplement: Supplementary file 1 [file Table1.docx]

Table S1: The primers and probes used for the methylation detection of the six genes

| DNA methylation primers | name | primer sequences（5’-3’） |
| --- | --- | --- |
| PAX1-1 | F | GCGGGATGTTGACGTGGTTC |
|  | R | TCCCGTATCGCCCACGAA |
|  | P | CCGAAATCCCGAACACGA |
| JAM3 | F | TTAGAAGGGACGGTGAGCG |
|  | R | TACCCCGAATACTATTTCCGAAT |
|  | P | GGCGAGGTAGAGTTTC |
| ZNF582 | F | CGTTGGTTTTATTATGGCGTAGTATT |
|  | R | GATAAATTCGGCGTATGAAA |
|  | P | CGAAAAATAACGTAAACGTA |
| PCDHGB7 | F | GAGTAGTGGATCGGTTTATATAGG |
|  | R | GAAAATTCGAACGAACGAGA |
|  | P | GAAAAACGCTGAACCGAC |
| SOX11 | F | GTTTGCGAGAGTTCGCGGTTC |
|  | R | TGACGATCGAAAGAACCCG |
|  | P | CGTAACTCGTACTGCAAACG |
| ZNF671 | F | GTTTTCGATTGAGACGTTTGTC |
|  | R | GGTGCGAAATCTGTTAACT |
|  | P | GAAAGCGCGGAGGAGATGT |
| GAPDH | F | TGTTTTGGGTGGTTATTGTGAA |
|  | R | CAATCCCTAACCCTACCTTTCTA |
|  | P | CCTTTCCTACTAAAACCCAAAACCAAAC |
| PAX1-2 | F | CGGGGTTATCGGGGGGA |
|  | R | CCGAACTACGCCAAACTACCG |
|  | P | AACGTGGTTAAGTATATTCGGGAT |

Table S2：Methylation of PAX1 or/and ZNF671 in different histopathology

| PAX1/ZNF671 | histopathology | | | | Total |
| --- | --- | --- | --- | --- | --- |
|  | inflammation | CIN1 | CIN2 | ≥CIN3 |  |
| **positive** | 0 | 6 | 6 | 79 | 91 |
| **negative** | 15 | 20 | 7 | 7 | 49 |
| **Total** | 15 | 26 | 13 | 86 | 140 |

Positive was defined as positive of either PAX1 or/and ZNF671, negative was defined as negative of both PAX1 and ZNF671.

Table S3：Methylation of PAX1 in different histopathology

| PAX1 | histopathology | | | | Total |
| --- | --- | --- | --- | --- | --- |
|  | inflammation | CIN1 | CIN2 | ≥CIN3 |  |
| positive | 0 | 5 | 4 | 77 | 91 |
| negative | 15 | 21 | 9 | 9 | 49 |
| Total | 15 | 26 | 13 | 86 | 140 |

Table S4: Methylation of ZNF671 in different histopathology

| ZNF671 | histopathology | | | | Total |
| --- | --- | --- | --- | --- | --- |
|  | inflammation | CIN1 | CIN2 | ≥CIN3 |  |
| positive | 0 | 3 | 4 | 69 | 91 |
| negative | 15 | 23 | 9 | 17 | 49 |
| Total | 15 | 26 | 13 | 86 | 140 |

Table S5: The diagnostic accuracies of different screening methods for CIN2+ lesions across different samples in HPV-negative population(n=75)

| Parameter | **Urine(n=25）** | **Cervical(n=28）** | **Vaginal(n=22)** | χ²/F | **P value** |
| --- | --- | --- | --- | --- | --- |
| Sensitivity | 0.00(0.00-43.84%) | 80.00% (49.02-94.33%) | 42.86% (15.11-74.87%) | 24.89 | **<0.001** |
| Specificity | 70.59% (45.73-88.19%) | 94.44% (72.71-99.86%) | 86.67% (61.22-97.18%) | 3.57 | 0.168 |
| PPV | 0.00(0.00-52.17) | 88.89% (51.75-99.72%) | 60.00% (23.04-88.84%) | 13.31 | **0.001** |
| NPV | 60.00% (40.65-77.34%) | 89.47% (67.28-97.68%) | 76.47% (52.48-91.15%) | 8.72 | **0.013** |
| PLR | 0.00(0.00-1.56) | 14.40(2.08-100.10) | 3.21(1.01-10.15) | - | - |
| NLR | 1.41(0.69-2.47) | 0.21(0.06- 0.69) | 0.66(0.35-1.24) | - | - |

Table S6: The diagnostic accuracies of different screening methods for CIN3+ lesions across different samples in HPV-negative population(n=75)

| Parameter | **Urine(n=25）** | **Cervical(n=28）** | **Vaginal(n=22)** | χ²/F | **P value** |
| --- | --- | --- | --- | --- | --- |
| Sensitivity | 0.00% (0.00-52.17%) | 100.00% (66.37-100.00) | 50.00% (15.71-84.29%) | 16.83 | **<0.001** |
| Specificity | 76.19% (54.95-90.13%) | 91.30% (0.7377-0.9819) | 86.96% (66.42-97.20%) | 2.68 | 0.261 |
| PPV | 0.00(0.00-52.17%) | 77.78% (45.26-95.09%) | 40.00% (12.11-73.73%) | 10.86 | **0.004** |
| NPV | 76.19% (54.95-90.13%) | 100.00% (86.23-100.00%) | 90.00% (71.33-98.79%) | 4.98 | 0.083 |
| PLR | 0.00(0.00-2.10) | 11.50(2.45-54.20) | 3.83(1.05-13.91) | - | - |
| NLR | 1.31(0.79-2.17) | 0.00(0.00-0.52) | 0.57(0.21-1.53) | - | - |
